# Effect of anti-C5 antibody on recuperation from ischemia/reperfusion-induced acute kidney injury

**DOI:** 10.1080/0886022X.2019.1677248

**Published:** 2019-10-29

**Authors:** Shani Zilberman-Itskovich, Ramzia Abu-Hamad, Moshe Stark, Shai Efrati

**Affiliations:** a Nephrology Division, Assaf-Harofeh Medical Center, Zerifin, Israel;; b Sackler School of Medicine, Tel-Aviv University, Tel-Aviv, Israel

**Keywords:** Acute kidney injury, rat model, ischemia-reperfusion injury, complement system, C5-antibody

## Abstract

**Aim:** The complement system is activated in acute kidney injury (AKI). Anti-C5 antibody targets the common terminal portion of the complement cascade that generate the terminal complex C5b-9 and has a renal-protective effect in paroxysmal nocturnal hemoglobinuria. However, the anti-C5 antibody’s role in ischemia/reperfusion (I/R)-induced AKI has not been fully investigated. We therefore evaluated its effect on the pathophysiological cascade of I/R-induced AKI.

**Methods:** Sprague–Dawley rats underwent unilateral right kidney nephrectomies with simultaneous clamping of the contralateral hilum for 60 min (ischemia), followed by reperfusion. In addition to a placebo, two treatment groups received either high or low doses of anti-C5 monoclonal antibody. After 48 h, the rats were euthanized, blood was drawn to evaluate systemic inflammation and to estimate glomerular filtration rate (GFR). The remaining kidney was removed for pathological evaluation and intra-renal complement activation.

**Results:** I/R induced significant intra-renal complement activation and systemic inflammation compared with unilateral nephrectomy group. The anti-C5 antibody ameliorated the intra-renal complement activation (intra-renal C3 and C6), reduced systemic inflammation (C-reactive protein, and systemic C3), decreased intra-renal acute tubular necrosis damage and improved GFR (seen by the sensitive marker, serum cystatin C; 1.63 mg/L (I/R + placebo), 1.36 mg/L (I/R + low dose) and 1.21 mg/L (I/R + high dose), *p* = .08 and .03 compared with I/R + placebo).

**Conclusion:** In I/R-induced AKI, the monoclonal anti-C5 complement factor ameliorates intra renal complement activation, decreases local and systemic inflammation and may improve GFR.

## Introduction

Acute kidney injury (AKI) is a common medical complication of up to 25% of intensive care unit admissions, with severe consequences, and mortality increasing up to 60% [1–3]. Frequently, AKI pathogenesis includes hypoxia that causes ischemia, followed by reperfusion [4,5]. The ischemia-reperfusion (I/R) process triggers intra-renal, but also systemic inflammation responses [6]. As part of the inflammatory response, the intra-renal complement system is activated [7]. Even though much knowledge has been gained regarding the post reperfusion pathophysiological cascade, there are currently no acceptable clinical therapeutic strategies that target at least one of the pathological cascade elements [8].

The complement system has an important role in the pathogenesis of renal failure, and is involved in glomerular, tubulointerstitial and vascular kidney injuries among others [9]. In humans, the complement system is involved in several types of AKIs including paroxysmal nocturnal hemoglobinuria, atypical hemolytic uremic syndrome, dense deposit disease and C3 glomerulopathy [10,11]. The anti-C5 antibody (Eculizumab) is clinically indicated for these conditions [10,11]. Animal models have demonstrated that complement receptors are overexpressed after reperfusion [12]. C3 and C6 knockout mice are relatively protected from renal I/R injury. However, this protection is missing in C4 knockout mice [13]. This indicates that the dominant part of the complement system involved in I/R injury is the alternative pathway [13]. The use of monoclonal antibody that prevent cleavage of C5 in an attempt to inhibit the effect of I/R injury in mice models has resulted in reduced inflammation and improved renal function [14]. However, an attempt to block C3 by the potent murine complement inhibitor CR1 related gene/protein y (Crry)-Ig did not demonstrate any beneficial effect in I/R-induced kidney injuries [15].

The aim of this study was to investigate the pathophysiological effects of the anti-C5 monoclonal antibody on an I/R-induced AKI rat model.

## Materials and methods

The study was approved by the Institutional Ethical Committee for Animal Experiments of Assaf-Harofeh Medical Center (approval number 62/2016) and was carried out in strict accordance with the recommendations of the National Institute of Health’s Guide for the Care and Use of Laboratory Animals. Forty-seven male Sprague–Dawley rats, 8 weeks old, weighing 250–300 g were used. The rats were housed in animal cages at a temperature of 25 °C with free access to food and water, in our institution’s animal facility. All surgeries were performed under halothane anesthesia, and all efforts were made to minimize suffering. Animals were designated to be euthanized after 48 h. Any change in animal behavior before that, including refusal to eat, drink or move, was addressed immediately and animals were euthanized.

### Ischemia-reperfusion model and treatment protocol

A total of 47 rats were assigned to one of the following groups: (1) unilateral nephrectomy followed by injection of saline 0.9% (sham + placebo, 6 rats); (2) unilateral nephrectomy followed by injection of 20 mg/kg rat anti-C5 antibody (sham + anti-C5, 5 rats); (3) ischemia-reperfusion procedure (I/R) followed by injection of saline 0.9% (I/R + placebo, 12 rats); (4) I/R followed by injection of 2 mg/kg rat anti-C5 antibody (I/R + low dose, 13 rats); (5) I/R followed by injection of 20 mg/kg rat anti-C5 antibody (I/R + high dose, 11 rats).

For the sham groups, only the high dose anti-C5 antibody was used to evaluate safety issues of administrating the treatment.

#### Surgical procedure

The rats were anesthetized with halothane under aseptic conditions. Unilateral nephrectomies were performed using a 4-cm long midline anterior approach laparotomy. After clamping the right renal hilum, the right kidney was removed. I/R was also performed using an anterior approach laparotomy and included a unilateral nephrectomy of the right kidney followed by 60 min of ischemia by clamping the left renal artery and vein. During these 60 min, the abdomen was closed. After 60 min of ischemia, the abdomen was reopened, the clamp was removed for reperfusion and the abdomen was closed in two layers (peritoneum and skin) [16]. At the end of the procedure, the rats received an intravenous injection through the tail vein before stopping halothane. Once recovered, the rats had free access to food and water. Urine was collected for evaluation using metabolic cages that collect all spontaneous urine to a separate container.

#### Anti-C5 antibody

The monoclonal antibody to the rat complement factor C5 was purchased from OAK Biosciences, CA. Antibody production was carried out uniquely for our specific study. The affinity of the antibody to C5 was evaluated according to the manufacturer protocol.

### Biochemical and pathological evaluations

The rats were euthanized under halothane anesthesia, 48 h following the procedure. Blood was drawn via a cardiac puncture, and the left kidney was removed for pathological evaluation. Whole blood was used for complete blood count using Beckman Coulter (DxH 800 Hematology Analyzer). The rest of the blood was centrifuged at 10,000*g* for 10 min and the serum was separated and tested for cystatin C, creatinine, urea, C-reactive protein (CRP), interleukin (IL)-1 and IL-6 as a marker of systemic inflammation, and C3 and C4 as classical and alternative pathway markers of anti-C5 efficacy.

Cystatin C was chosen as the marker of choice for AKI since it is a more sensitive marker than creatinine and urea for estimation of the glomerular filtration rate (GFR) in an AKI setting [17]. Cystatin C was measured by a particle-enhanced immunoturbidimetry method, with a commercially available Dako Cystatin C PET Reagent Set (DAKO, Hamburg, Germany). Creatinine, urea and CRP were estimated on COBAS 8000 autoanalyzer (ROCHE Diagnostics, Indianapolis, IN).

The classical and alternative complement pathways (HBT, Uden, The Netherlands, classical complement pathway, Rat, Assay, CAT: HIT 410; HBT, Uden, The Netherlands, alternative complement pathway, Rat.cat: HIT 412), C3 and C4 (ICL, Portland, OR, Rat C3 ELISA cat: E-25C3; MYBIOSOURSE-MYBIO, San Diego, CA, C4 ELISA kit: Rat complement 4, C4 ELISA kit MBS70336) were assessed by specific ELISAs according to the manufacturer’s instruction. Data are presented in percentage from maximal complement activity possible for the specific test.

IL-1 and IL-6 were assessed by specific ELISAs (R&D Systems, Minneapolis, Minnesota) according to the manufacturer’s instructions.

### Pathological evaluation

Kidneys were preserved in 4% formalin and subsequently embedded in paraffin [16,18]. Paraffin-embedded slides were prepared by a standard procedure. One slide from each rat was stained with hematoxylin and eosin dye for histopathologic examination under a light microscope. Another slide from each rat was used for immunofluorescence staining procedures, for evaluating the mouse anti-rat complement C3 (NOVUS, Centennial CO) and rabbit anti-C6, complement component 6 (Proteintech, Rosemont, IL), according to manufacturer’s instructions.

To quantify the complement C3 and C6, we used the same immunofluorescence described above and quantifying the staining using ‘Lionheart FX Automated Live Cell Imager’ software (BioTek, Winooski, VT) on the same slides.

Computerized morphometry was performed on an Olympus CKX 41 microscope using the CMS-2-M system as part of the Advanced Measurement Systems, Ltd. (Israel). The system included a digital color CCD camera (1600 × 1200 pixels) and a software package for pathology and immunofluorescence evaluation.

Tubular necrosis was identified and calculated as the percentage of damaged tubules from the total of all tubules in the examined kidney [16,18]. A cross-section of the entire left kidney was used for evaluation, and all tubules were evaluated. In addition, nucleus degeneration and proliferation were evaluated and presented as percentage of tubules involved per total tubule count. All pathological evaluations were calculated as percentage of the tubules involved from total tubules in the kidney’s cross section.

### Statistical analysis

Statistical analysis was carried out using SPSS (version 20; IBM, Armonk, New York, NY) software. Residuals were first tested for normal distributions (Shapiro–Wilk test) and equality of variance (Levene’s test). Nonparametric tests were used where appropriate. Group comparisons were calculated using Student’s independent *t*-test and analysis of variance for parametric variables. A Kruskal Wallis test was used for nonparametric variables. The significance level was set to *p* < .05. Values are given as means ± standard deviation (SD).

## Results

### Safety and effectivity of anti-C5 administration

Administration of the high anti-C5 dosage to the sham group was found to be safe with no significant changes as compared to the sham + placebo group with respect to blood count, renal function tests, CRP, complement cascade and kidney histological and immunofluorescence findings (Table 1). Cystatin C was 0.98 ± 0.34 and 0.77 ± 0.84 for sham + placebo and sham + anti-C5, respectively (*p* = .62). CRP was 0.33 ± 0.06 and 0.3 ± 0.01 for sham + placebo and sham + anti-C5, respectively (*p* = .19).

**Table 1.** Blood and urine tests evaluation of nephrectomy and ischemia/reperfusion groups.

	Sham + placebo	Sham + anti-C5 (*p* value compared with sham + placebo)	I/R + placebo (*p* value compared with sham + placebo)	I/R + low dose (*p* value compared with I/R + placebo)	I/R + high dose (*p* value compared with I/R + placebo)
*Blood tests*					
Hemoglobin (g/dL)	13.2 ± 0.8	12.8 ± 0.4 (*p*=.48)	11.8 ± 1.3 (*p*=.08)	12.2 ± 1 (*p*=.29)	11.7 ± 0.9 (*p*=.89)
White blood cells (K/uL)	4.5 ± 2.9	5.5 ± 3.3 (*p*=.28)	4.4 ± 3.1 (*p*=.92)	6 ± 3.3 (*p*=.46)	3.8 ± 3.6 (*p*=.65)
Platelets (K/uL)	698 ± 213	650 ± 299 (*p*=.78)	740 ± 225 (*p*=.6)	765 ± 93 (*p*=.82)	884 ± 171 (*p*=.016)
*Renal function tests and histopathology*					
Cystatin C (mg/L)	0.98 ± 0.34	0.77 ± 0.84 (*p*=.62)	1.63 ± 0.62 **(*p*=.04)**	1.36 ± 0.71 (*p*=.08)	1.21 ± 0.46 **(*p*=** .**03)**
Acute tubular necrosis (%)	0 ± 0	0 ± 0 (*p*>.99)	37.5 ± 34.5 **(*p*=** .**045)**	18.8 ± 12.5 (*p*=.26)	25 ± 20.4 (*p*=.43)
Nucleus degeneration (%)	0 ± 0	0 ± 0 (*p*>.99)	25 ± 5 **(*p*=** .**01)**	25 ± 28.9 (*p*>.99)	17.9 ± 18.9 (*p*=.38)
Proliferation (%)	0 ± 0	0 ± 0 (*p*>.99)	33.3 ± 20.4 **(*p*=**.**01)**	31.3 ± 23.9 (*p*=.89)	35.7 ± 34.9 (*p*=.89)
Protein casts- cortex (%)	0 ± 0	0 ± 0 (*p*>.99)	8.3 ± 12.9 (*p*=.18)	6.3 ± 12.5 (*p*=.81)	10.7 ± 19.7 (*p*=.81)
Protein casts- medulla (%)	0 ± 0	0 ± 0 (*p*>.99)	25 ± 22.4 **(*p*=**.**04)**	18.8 ± 23.9 (*p*=.68)	32.1 ± 31.3 (*p*=.65)
*Immunofluorescence*					
C3 (intensity* 10^9^)	0.6 ± 0.27	0.52 ± 0.25 (*p*=.67)	3.03 ± 0.57 **(*p*<**.**001)**	1.46 ± 0.57 **(*p*=**.**002)**	2.31 ± 0.28 **(*p*=**.**05)**
C6 (intensity* 10^9^)	1.03 ± 0.48	1.36 ± 0.03 (*p***=**.16)	2.68 ± 0.52 **(*p*<**.**001)**	1.4 ± 0.11 **(*p*=**.**002)**	1.45 ± 0.52 **(*p*=**.**003)**
*Systemic inflammatory tests (serum)*					
CRP (mg/L)	0.33 ± 0.06	0.3 ± 0.01 (*p*=.19)	0.47 ± 0.21 **(*p*=**.**05)**	0.36 ± 0.1 **(*p*=**.**013)**	0.33 ± 0.06 **(*p*=**.**01)**
IL-1 (pg/mL)	109 **±** 3	129 ± 35 (*p*=.57)	117 ± 6 (*p*=.21)	128 ± 50 (*p*=.48)	114 ± 6 (*p*=.33)
IL-6 (pg/mL)	102 **±** 29	119 **±** 48 (*p*=.58)	87 ± 4 (*p* = 58)	94 ± 13 (*p*=.07)	88 ± 7 (*p*=.72)
C3 (µcg/L)	68.8 ± 24.8	(N/A)	45.5 ± 47.1 (*p*=.44)	3.3 ± 1.9 **(*p*=**.**03)**	22.0 ± 9.3 (*p*=.36)
C4 (ng/L)	287 ± 76	(N/A)	371 ± 86 (*p*=.16)	468 ± 108 (*p*=.1)	383 ± 125 (*p*=.85)
Classical (%)	76 ± 5	(N/A)	73 ± 12 (*p*=.61)	81 ± 9 (*p*=.17)	72 ± 13 (*p*=.86)
Alternative (%)	85 ± 9	(N/A)	79 ± 25 (*p*=.57)	84 ± 14 (*p*=.67)	81 ± 9 (*p*=.88)

Data are presented as mean ± SD.

*p*-value <.05 is highlighted in bold.

I/R: ischemia reperfusion; CRP: C-reactive protein; IL: interleukin.

With regards to activity, intra-renal C6 was significantly reduced in both low and high dose treatment groups. This means that the used dosages of anti-C5 antibody were effective in blocking the intra-renal complement cascade (Table 1).

### I/R-induced AKI, inflammation and complement activation

**Figure 1.**
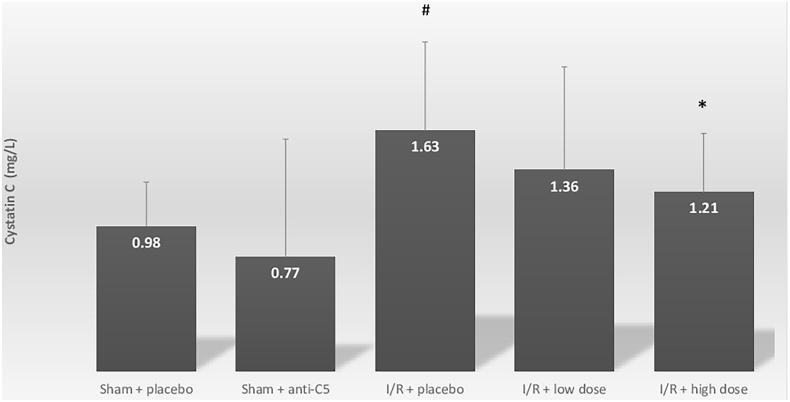
Serum cystatin C levels for sham and ischemia-reperfusion groups. Serum cystatin C levels are in milligram per liter for all groups. Group comparisons were calculated using Student’s independent *t*-test. A comparison was conducted between both the sham groups: sham + placebo and I/R + placebo and between I/R + placebo and I/R + treatment groups. Data shown are mean ± SD. *p < .05 for I/R + placebo group versus I/R + treatment group. #*p* < .05 for I/R + placebo group versus Sham + placebo group. I/R: ischemia-reperfusion.

**Figure 2.**
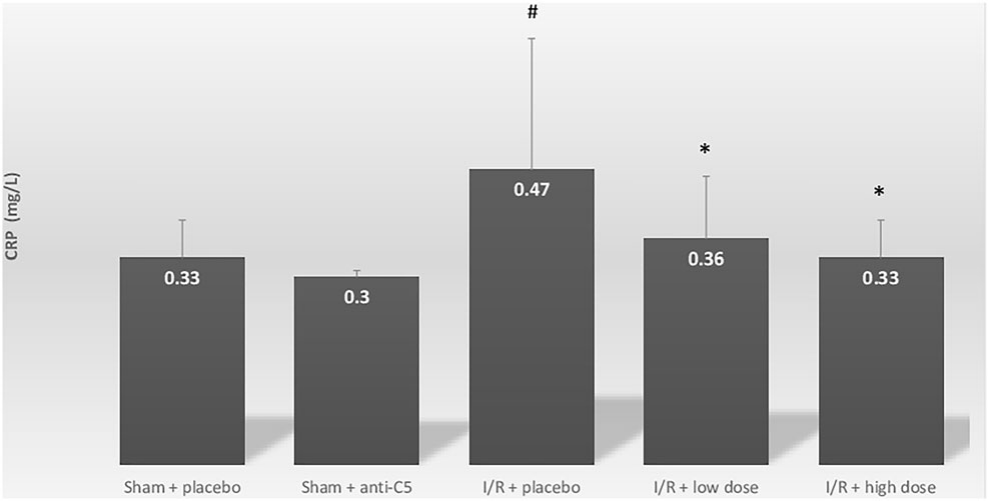
Serum C-reactive protein levels for sham and ischemia-reperfusion groups. Serum CRP levels are in milligram per liter for all groups. Group comparisons were calculated using Student’s independent *t*-tests. A comparison was conducted between both the sham groups: sham + placebo and I/R + placebo and between I/R + placebo and I/R + treatment groups. Data shown are mean ± SD. *p < .05 for I/R + placebo group versus I/R + treatment group. #*p* < .05 for I/R + placebo group versus Sham + placebo group. I/R: ischemia-reperfusion; CRP: C-reactive protein.

**Figure 3.**
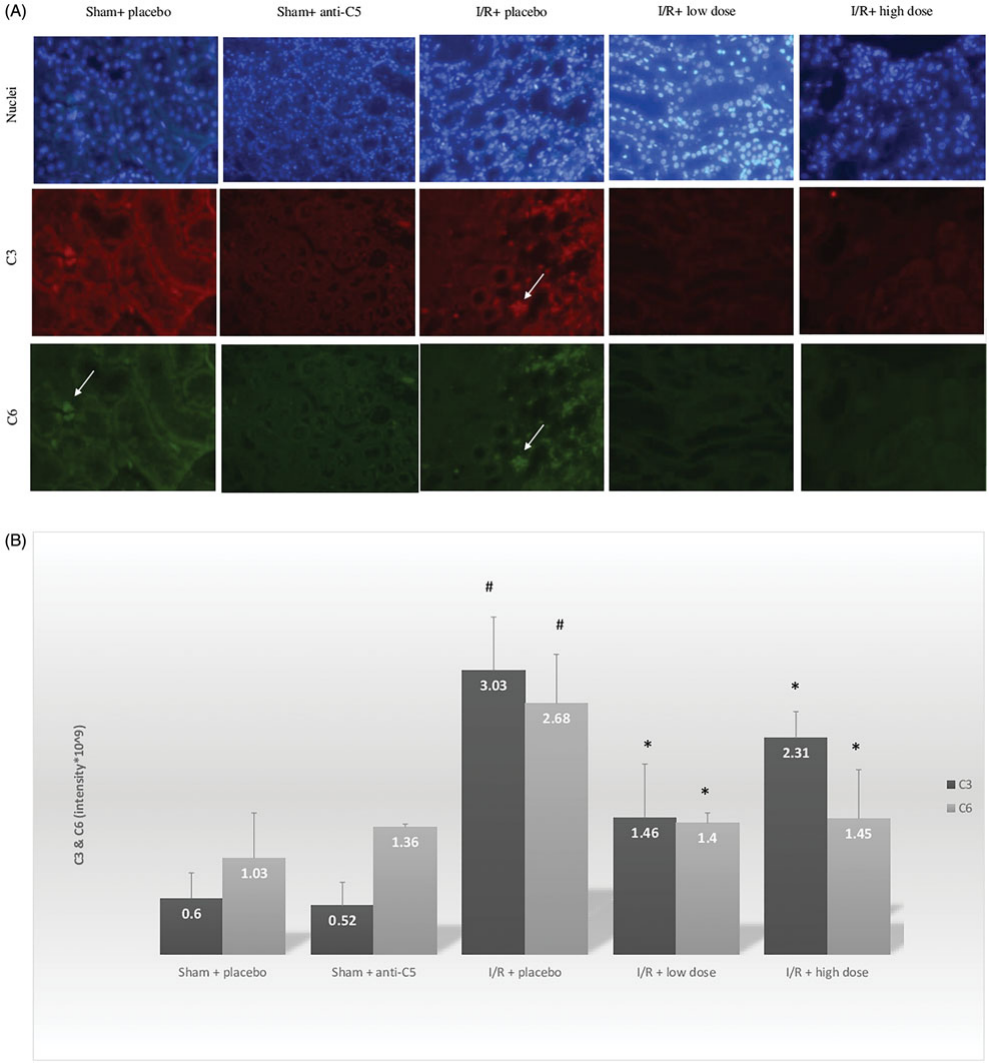
Renal immunofluorescence staining for C3 and C6 in the sham and ischemia/reperfusion groups treated by placebo or anti-C5 antibody. (A) Immunofluorescence of C3 and C6 of sham and I/R groups. Nucleolus is seen in blue. Arrows pointing to immune deposits in tubular cells. (B) Intensity evaluation of C3 and C6 immunofluorescence. *p<.05 for I/R + placebo group versus I/R + treatment group. #*p*<.05 for I/R + placebo group versus Sham + placebo group. I/R: ischemia-reperfusion.

Compared with the sham + placebo group, the I/R group demonstrated an increase in serum cystatin C, from 0.98 ± 0.34 to 1.63 ± 0.62 mg/L, *p* = .04 (Figure 1). As expected, histologic evaluations revealed significant acute tubular necrosis (ATN) and proliferation in the I/R + placebo group, and no ATN in the sham + placebo group. In addition to AKI, I/R induced systemic inflammation, marked by increases in CRP levels from 0.33 ± 0.06 mg/L to 0.47 ± 0.21 (*p* = .05) (Table 1; Figure 2). Intra-renal complement activation, marked by immunofluorescence intensity, revealed increased renal deposits of C3 and C6 in the I/R + placebo group compared with sham + placebo (Table 1; Figure 3).

### I/R + anti-C5

#### Estimated GFR

Serum cystatin C improved in a dose-dependent manner in the I/R + anti-C5-treated groups: 1.63 ± 0.62 mg/L, 1.36 ± 0.71 mg/L and 1.21 ± 0.46 mg/L in the I/R + placebo, I/R + low dose I/R + high dose anti-C5 groups, respectively (*p*=.08 and .03 as compared to placebo, respectively) (Figure 1).

#### Systemic inflammation and systemic complement Cascade

Systemic inflammation, marked by serum CRP, decreased with anti-C5 antibody administration in a dose-dependent manner. CRP values were 0.47 ± 0.21, 0.36 ± 0.1 and 0.33 ± 0.06 mg/L for I/R + placebo, IR + low dose anti-C5 and IR + high dose anti-C5 groups, respectively (*p*=.013 and .01 for I/R + placebo vs. treatment groups, respectively) (Figure 2). In addition, serum C3 levels were reduced by the anti-C5 antibody from 45.5 ± 47.1 to 3.3 ± 1.9 and 22.0 ± 9.3 µcg/L in IR + placebo, IR + low and high dose (*p=* .03 and .36 for placebo vs. treatment groups, respectively). IL-1 and IL-6 did not reveal significant differences between the groups (Table 1).

#### Intra-renal C3 and C6 infiltrates

The complement factors C3 and C6 are both related to C5 activation [19]. Treatment with the anti-C5 antibody significantly decreased the intra-renal infiltrates of both C3 and C6 surrounding the tubules, both in the cortex and the medulla, compared with the IR + placebo group. As can be seen in Figure 3, the intra-renal infiltrates of C6 decreased significantly.

#### Renal pathology

**Figure 4.**
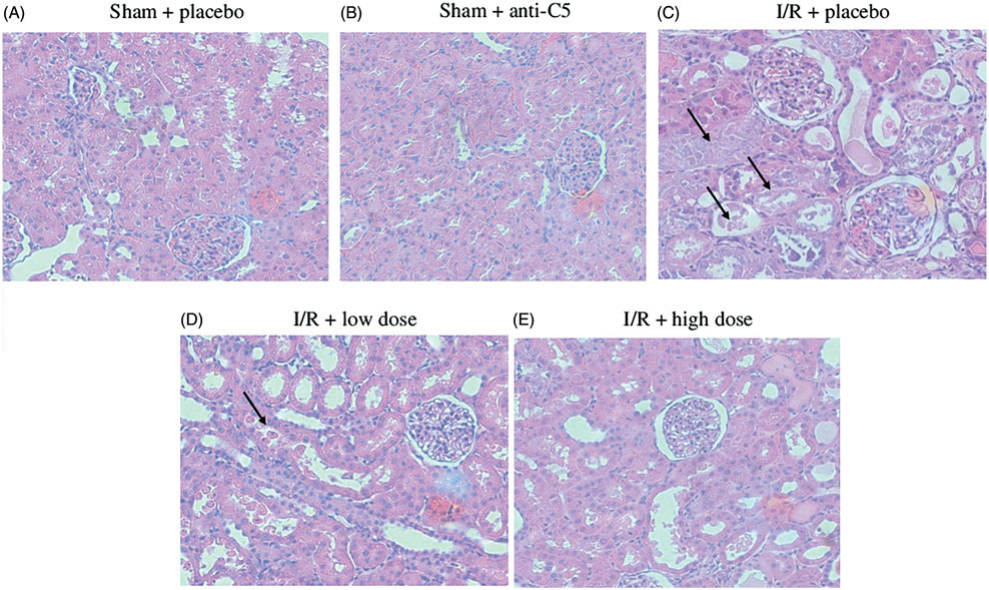
Renal pathology in different study groups. Hematoxylin and eosin staining for sham and I/R groups. Arrows point to tubular necrosis and damage. Hematoxylin and eosin staining of renal biopsy for sham and after I/R. All histologic slides show preserved glomeruli, without specific glomerular pathology. Tubular damage is the prominent feature with severe tubular necrosis for I/R + placebo, manifested as tubular cells detached from the tubular structure, and loss of the cell nucleus. Tubular cells in both treatment groups are less prominent. In the higher dose of I/R + 20 mg, tubules demonstrate hyaline casts inside tubular lumen. I/R: ischemia reperfusion.

The findings of the pathological evaluation are summarized in Table 1 and Figure 4. Treatment with anti-C5 trended toward a decrease in the ATN damage from 37.5 ± 34.5%, in the IR + placebo group, to 18.8 ± 12.5% in the I/R + low dose anti-C5 group and 25 ± 20.4% in the I/R + high dose anti-C5 group. Nucleus degradation was decreased in the high anti-C5 group from 25 ± 5% to 17.9 ± 18.9%. There was no significant change in proliferation between I/R groups.

## Discussion

We investigated the effect of the anti-C5 antibody on I/R-induced AKI. I/R induced significant intra-renal complement activation and systemic inflammation. The anti-C5 antibody ameliorated the expected intra-renal complement activation, reduced the systemic inflammation and decreased the intra-renal ATN damage. In addition, eGFR estimated by serum cystatin C, a sensitive marker, was significantly improved.

The pathophysiological cascade of I/R-induced AKI correlates with the immune system activation, mostly the innate immune system [6,20]. The complement system, as part of the immune system, is also involved [9]. The complement system is primary in causing the injury and it can be induced by other elements of the immune system [21,22]. The complement system can be activated by three main pathways, the classical pathway, the alternative pathway and the mannose binding lectin pathway [7]. I/R injury activation of the complement system is not unique to the kidney. Other organs such as the heart, intestine, liver and skeletal muscle have similar involvements [7]. In this study, I/R induce AKI, produced significant intra-renal complement activation and systemic inflammation. The systemic inflammation (measured by CRP levels) and intra-renal activation of the complement cascade were both reversed by the anti-C5 antibody. Even though the systemic and the intrarenal inflammations were inhibited with anti-C5 treatment, there was no major systemic effect on the circulatory complement C4, classic or alternative complement pathways. Circulatory complement C3, which is the common pathway of all three complement activation pathways was reduced with treatment.

Anti-C5 prevents C5b-9 complex generation but it can also ameliorate the generation of C5a. One possible explanation for the reduction of the systemic inflammation (marked by CRP) is the effect of the complement product C5a on the immune system. C5a, in addition to C3a, is an important anaphylactic and chemotactic factor involved in apoptosis and inflammation [22,23]. There are several ways to suppress the immune response, including corticosteroid treatments and several types of chemotherapies [24]. However, extensive suppression of the immune system can be hazardous since it exposes the patients to higher risks of sepsis. In addition, at least certain elements of the immune system play important roles in the needed regenerative processes [24]. Accordingly, new alternatives that target specific elements of the immune system are being investigated. Eculizumab, a monoclonal antibody against the C5 complement protein, is currently used in clinical renal injury settings, mainly for atypical hemolytic uremic syndrome (a-HUS), PNH and in C3 glomerulopathy [25–29]. However, the data regarding the beneficial effect of blocking the complement system in the setting of I/R-induced AKI is conflicting. Beneficial outcome results were observed in a mouse model of AKI, using mouse anti-C5 antibody [14]. However, a study that attempted to block C3 activation using a knockout model did not demonstrated a beneficial effect [15]. In other studies, C3 activation blockage using small interfering RNAs, or an anti-C1 antibody, improved renal outcomes [30].

In this study, the effect of a rat anti-C5 antibody was investigated in the setting I/R-induced AKI. The treatment was given in two doses and both doses successfully reduced the complement infiltrates in the I/R kidneys. Both final complement common pathways (detected by C6) and C3 levels were reduced in immunofluorescence staining, a finding that demonstrates the intensifying effect that the complement system reflects on itself.

The use of complement target antibodies cannot abolish the hypoxia-related damage that was already done to the renal tubule. Therefore, the use of the anti-C5 antibody does not completely reverse the kidney damage. Nevertheless, it can and did reduce the post-ischemic reperfusion injury that is related to over-activation of the inflammatory cascade. The expected intra-renal ATN damage was reduced and the estimated GFR (using the sensitive cystatin C marker) was also improved. However, attention should be given to the histological findings of higher percentages of hyaline casts in the anti-C5 treated groups. The hyaline casts might be related to a very high protein load filtered through the glomeruli, a process similar to cast nephropathy in multiple myeloma [31]. While preparing the anti-C5 antibody for injection, it is heated from below zero to +25 °C and this process can cause some protein degradation. Degradation can also occur after injection, while in the circulation, and the degraded immunoglobulins (peptides) can accumulate in the tubules of the injured kidneys as in multiple myeloma related casts nephropathy which can further exacerbate the renal damage.

## Conclusions

In conclusion, treatment with anti-C5 antibody in the setting of I/R-induced AKI can block the intra-renal complement activation, reduce systemic inflammation, improve the extent of tubular damage and improve eGFR. Based on these results, we have shown that the complement system has a role in the pathogenesis of I/R-induced AKI.
